# Unveiling the enzymatic pathway of UMG-SP2 urethanase: insights into polyurethane degradation at the atomic level†

**DOI:** 10.1039/d4sc06688j

**Published:** 2024-12-18

**Authors:** P. Paiva, L. M. C. Teixeira, R. Wei, W. Liu, G. Weber, J. P. Morth, P. Westh, A. R. Petersen, M. B. Johansen, A. Sommerfeldt, A. Sandahl, D. E. Otzen, P. A. Fernandes, M. J. Ramos

**Affiliations:** a LAQV@REQUIMTE, Departamento de Química e Bioquímica, Faculdade de Ciências, Universidade do Porto Rua do Campo Alegre s/n 4169-007 Porto Portugal mjramos@fc.up.pt; b EnZync Center for Enzymatic Deconstruction of Thermoset Plastics; c Junior Research Group Plastic Biodegradation, Department of Biotechnology & Enzyme Catalysis, Institute of Biochemistry, University of Greifswald Felix-Hausdorff-Str. 8 17489 Greifswald Germany; d Tianjin Institute of Industrial Biotechnology, Chinese Academy of Sciences 32 West Seventh Avenue, Tianjin Airport Economic Area Tianjin 300308 China; e Macromolecular Crystallography, Helmholtz-Zentrum Berlin Alber-Einstein-Straße 15 12489 Berlin Germany; f Department of Biotechnology and Biomedicine, Technical University of Denmark Søltofts Plads DK-2800 Kongens Lyngby Denmark; g Teknologisk Institut Kongsvang Alle 29 DK-8000 Aarhus Denmark; h Interdisciplinary Nanoscience Center (iNANO). Aarhus University Gustav Wieds Vej 14 DK-8000 Aarhus Denmark

## Abstract

The recently discovered metagenomic urethanases UMG-SP1, UMG-SP2, and UMG-SP3 have emerged as promising tools to establish a bio-based recycling approach for polyurethane (PU) waste. These enzymes are capable of hydrolyzing urethane bonds in low molecular weight dicarbamates as well as in thermoplastic PU and the amide bond in polyamide employing a Ser-Ser_*cis*_-Lys triad for catalysis, similar to members of the amidase signature protein superfamily. Understanding the catalytic mechanism of these urethanases is crucial for enhancing their enzymatic activity and improving PU bio-recycling processes. In this study, we employed hybrid quantum mechanics/molecular mechanics methods to delve into the catalytic machinery of the UMG-SP2 urethanase in breaking down a model PU substrate. Our results indicate that the reaction proceeds in two stages: STAGE 1 – acylation, in which the enzyme becomes covalently bound to the PU substrate, releasing an alcohol-leaving group; STAGE 2 – deacylation, in which a catalytic water hydrolyzes the enzyme:ligand covalent adduct, releasing the product in the form of a highly unstable carbamic acid, expected to rapidly decompose into an amine and carbon dioxide. We found that STAGE 1 comprises the rate-limiting step of the overall reaction, consisting of the cleavage of the substrate's urethane bond by its ester moiety and the release of the alcohol-leaving group (overall Gibbs activation energy of 20.8 kcal mol^−1^). Lastly, we identified point mutations that are expected to enhance the enzyme's turnover for the hydrolysis of urethane bonds by stabilizing the macrodipole of the rate-limiting transition state. These findings expand our current knowledge of urethanases and homolog enzymes from the amidase signature superfamily, paving the way for future research on improving the enzymatic depolymerization of PU plastic materials.

## Abstract 2

This work uncovers the detailed catalytic mechanism of UMG-SP2 urethanase, offering insights into improving PU degradation.

## Graphical abstract

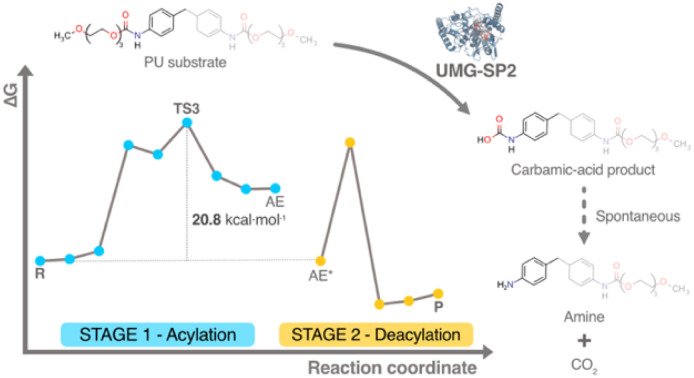

## Introduction

Plastics have become fundamental to modern society, due to their durability, versatility, and low production cost. This widespread reliance on plastics permeates nearly every industrial sector, including packaging, construction, electronics, and beyond.^1^ Consequently, global plastic production has significantly increased over the past 50 years. A combination of widespread use and poor end-of-life planning have led to the accumulation of these recalcitrant materials throughout the environment.^2^ In 2022, <10% of all globally produced plastic was recycled.^3^ This scenario now poses serious threats to both the environment and human health.^4,5^ Therefore, there is an urgent need for the development of efficient, sustainable, and economically viable solutions to address the end-of-life of these materials.^6^

Polyurethanes (PU) are among the most widely used types of recalcitrant plastics, due to their broad scope of properties. PU accounts for 5.3% of the 400 million tons of plastic produced globally each year, placing it sixth in terms of synthetic polymer production.^3^ The carbamate bond, commonly referred to as the urethane bond in polyurethane chemistry, R_1_OC=OCNR_2_R_3_, is formed by combining an isocyanate (typically methylene diphenyl diisocyanate or toluene diisocyanate) with a polyol (*e.g.*, polyethers and polyesters). Depending on the formulation, polyurethane materials can be either thermoplastic or thermoset polymers, allowing for a wide range of applications, including adhesives, coatings, foams, elastomers, and sealants.^7^

In 2018, hard and flexible foams constituted 68% of the PU market share, indicating that most of the applications used thermoset PU.^8^ Unlike thermoplastics, thermoset PUs have highly cross-linked structures and are thus insoluble in both water and organic solvents. Thermosets cannot be repeatedly melted and reshaped upon heating, but only thermally decomposed through processes like pyrolysis or chemical depolymerization at very high temperatures.^7,9^ Consequently, recycling thermoset PUs is limited to grinding, adhesive bonding, or chemical methods such as glycolysis. The first two are secondary recycling processes, which do not depolymerize the waste but simply repurpose the recycled polymer for less demanding applications. At the same time, the third option is a tertiary recycling method that molecularly disassembles the waste polymers and transform them to produce other chemicals.^10^ The current options for thermoset PU recycling are far from satisfactory, as the mechanically-recycled polymers can only be used for alternative applications (*e.g.*, as fillers) with significantly reduced market value, or the chemical depolymerization process consumes high amounts of energy (*ca.* 817 kg CO_2_-eq. per t PU waste).^11,12^ Therefore, developing a more efficient and environmentally friendly recycling method is also economically beneficial.

Enzymatic depolymerization is one of the most promising strategies for addressing the end-of-life of recalcitrant hydrolyzable plastics.^6,13,14^ Unlike chemical recycling, the enzymatic process does not require harsh conditions (*e.g.*, high temperatures and/or of toxic compounds). Still, it can suffer from low catalytic efficiency provided by the native biocatalysts. Since Müller *et al.* reported the first PET hydrolase (PETase) in 2005,^15^ an enzyme capable of depolymerizing polyethylene terephthalate (PET) into its monomers including terephthalate and ethylene glycol, there has been a rise in interest in discovery and design more powerful PETases.^16–19^ Building on this, the company Carbios engineered an enzyme that hydrolyzes around 90% of pretreated post-consumer PET waste within 10 hours, demonstrating the potential of enzymatic depolymerization for industrial recycling applications.^20,21^

The commercial success of enzymatic PET recycling has sparked renewed interest in biocatalytic recycling of other mass-produced plastics, with PU emerging as a logical next target due to its hydrolyzable backbone linked by carbamate bonds. The history of searching for PU-degrading microorganisms and enzymes is not necessarily shorter than that for polyesters; nonetheless, the results have been less promising, as most reported enzymes are polyester hydrolases that are exclusively active on polyester-based PU.^7,22,23^ In 2023, Branson *et al.*^24^ discovered three urethanase enzymes (UMG-SP1, UMG-SP2, and UMG-SP3), and demonstrated their effectiveness in PU recycling *via* a two-step chemoenzymatic process, achieving complete conversion of post-consumer soft foam waste composed of toluene diisocyanate (TDI)-based polyether-PU into the respective polyols and aromatic diamines. These enzymes were identified through a metagenomic screening from an isolated soil sample exposed to PU-related chemicals for a long period. Remarkably, UMG-SP2 hydrolyzed more than 90% of the low molecular weight dicarbamate TDI-diethylene glycol within 24 h.

The crystal structure of UMG-SP1, which shares 52.4% sequence identity with UMG-SP2, was recently solved along with a series of characterizations and engineering, demonstrating its depolymerization activity on pretreated polyamide and thermoplastic PU.^25^ Simultaneously, we elucidated ligand-free and ligand-bound crystal structures of UMG-SP2 and validated its depolymerization ability on PU polymer in a separate study.^26^ Consequently, our research established a robust foundation for enhancing UMG-SP2's catalytic efficiency to fulfill the demands of its applications in industrial PU recycling. To achieve this goal, understanding the structure-to-activity relationship will allow us to establish the catalytic mechanism of UMG-SP2 and subsequently identify “prejudicial residues” that destabilize the rate-limiting transition state (TS) in relation to the reactant state. Such undesirable residues increase the reaction activation energy and consequently decrease the rate, making them the most promising targets for mutations aimed at enhancing UMG-SP2's catalytic efficiency rationally.

**Scheme 1:**
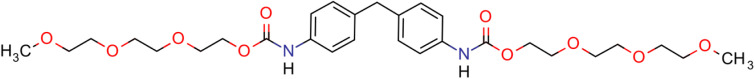
The chemical structure of the substrate used in this work to mimic a PU segment, di-urethane ethylene 4,4′-methylenedianiline (DUE-MDA).

UMG-SP2 belongs to the amidase signature superfamily, which has a highly conserved active site. Most members of this superfamily share the Ser_nuc_-Ser_*cis*_-Lys catalytic triad, including UMG-SP2 (Ser190_nuc_-Ser166_*cis*_-Lys91).^27–29^ In this type of catalytic triad, the mechanism typically starts with a proton transfer from Ser_*cis*_ to Lys, enabling the remaining Ser_nuc_ residue to perform a nucleophilic attack on the substrate's amide bond. For this to happen, the catalytic Lys must adopt a neutral state, as only in this state can it accept a proton donated by the Ser_*cis*_ residue. Later in the reaction, Lys returns the proton to Ser_*cis*_, which in turn transfers its proton to the substrate's leaving group, culminating in the formation of the acyl-enzyme state and the release of an amine. To regenerate the enzyme, a water molecule enters the active site and conducts a nucleophilic attack on the substrate's carbonyl carbon that is bound to the Ser_nuc_. Consequently, the Ser_nuc_ residue becomes deacylated, releasing the product as a carboxylic acid, and the enzyme regenerates for a new catalytic cycle.^30^ Even though the typical mechanism for Ser_nuc_-Ser_*cis*_-Lys has been studied before, the atomistic and energetic details vary throughout the family members. Additionally, to the best of our knowledge, the hydrolysis of the urethane bond catalyzed by this type of enzyme has not been elucidated. Thus, it is essential to study the catalytic mechanism of urethanases in detail, instead of relying on general studies for the amidase family. Therefore, we set out to establish the mechanism of the urethane bond hydrolysis catalyzed by UMG-SP2 in order to propose mutations to enhance its efficiency. For this purpose, we used the symmetric di-urethane ethylene 4,4′-methylenedianiline (DUE-MDA) as a substrate (Scheme 1).

This compound was chosen because it is a dicarbamate with two of the most often employed structural compositions (MDA as the isocyanate and polyether-based polyol) in industrial PU monomer formulations. We employed an adiabatic quantum mechanics/molecular mechanics methodology (QM/MM). Our calculations provided a detailed atomistic and energetic description of the mechanism and identified potential productive mutations to improve UMG-SP2's catalytic efficiency for urethane bond hydrolysis.

## Methods

### UMG-SP2 structure preparation

We assembled the computational model used to explore the reaction mechanism of UMG-SP2 from the recently discovered X-ray structure of UMG-SP2 complexed with phenylmethanesulfonyl fluoride^26^ (PDB ID: 8WDW, 2.16 Å resolution). From this structure, we selected chain A and all crystallographic water molecules located within 10 Å of this chain (a total of 439 residues and 272 water molecules) to be included in the model.

We estimated the protonation state of all residues, at pH 8.0, with the empirical p*K*_a_ predictor PROPKA 3.5.0 (Table S1†).^31^ PROPKA predicted that the side chain of the catalytic triad Lys91 should exist mostly in its neutral form (–NH_2_). Visual inspection revealed that Lys91, buried in the active site cavity and therefore not solvated by water, was not close to any negatively charged residue (*i.e.*, Asp or Glu) that could stabilize a hypothetical positive state of this residue, thus explaining why PROPKA estimated a p*K*a value of 6.09. In its neutral form, the side chain of Lys91 is able to donate two hydrogen bonds to two nearby serine residues (Ser167 and Ser185, located at ≈3.0–3.1 Å) and simultaneously accept a hydrogen bond from the catalytic Ser166_*cis*_ (2.35 Å). We further analyzed visually the active site residues and other titratable residues in their surroundings to thoroughly verify the selected protonation states. Among the nine existing histidine residues, six were N_ε_-protonated (His159, His196, His215, His271, His376, and His439), and three were N_δ_-protonated (His108, His111, and His159).

### Docking of the PU substrate and parameterization of the UMG-SP2:DUE-MDA complex

Given that UMG-SP2 hydrolyzes low molecular weight dicarbamates of aromatic diamines (*e.g.*, toluene-2,4-diamine (TDA) and 4,4′-methylenedianiline (MDA) derivatives),^24^ we decided to study the catalytic mechanism of this enzyme using the symmetric DUE-MDA compound. This ligand exhibits two urethane bonds on each side of the MDA core, each attaching a triethylene glycol monomethyl chain (Scheme 1). We employed the GaussView 5.0 software^32^ to build the molecular structure of DUE-MDA, which was subsequently optimized with the Gaussian 09 software^33^ at the HF/6-31G(d) level of theory. Then, the DUE-MDA ligand was docked to the active site of UMG-SP2 using the molecular docking software GOLD (Genetic Optimization Ligand Docking).^34^ The binding region was defined as a 10 Å radius sphere centered on the O_γ_ atom of the catalytic Ser190_nuc_. We carried out the docking procedure using GOLD's automatic genetic algorithm settings with the search efficiency set to 100%, and the results were scored using the CHEMPLP fitness function.^35^ We found it advantageous to apply distance constraints to ensure an adequate positioning of the target urethane group regarding the active site residues. Specifically, the distances between the carbonyl oxygen of the urethane group and the NH_backbone_ groups of Ile187 and Gly188 were forced to lie within 1.5 and 3.0 Å (spring constant of 5.0). The docking procedure finished when the top three solutions laid within 1.5 Å RMSD (Root Mean Square Deviation) of each other. 10 different solutions were obtained, and we further performed a detailed analysis of the interatomic distances, hydrogen bonds, and close contacts between the docked ligand and the active site residues. In the end, we selected the solution that simultaneously exhibited the shortest O_γ_(Ser190_nuc_)–C_carbonyl_(DUE-MDA), O_carbonyl_(DUE-MDA)–NH_backbone_(Ile187), and O_carbonyl_(DUE-MDA)–NH_backbone_(Gly188) distances, to pursue the calculations.

### Molecular dynamics simulations

We neutralized the enzyme:ligand complex obtained from the docking procedure with 17 Na^+^ counterions and placed it in the center of a rectangular box of TIP3P water molecules,^36^ whose faces were at least 12 Å away from the enzyme's surface. This was accomplished by using the XLEaP module of AMBER 18.^37^ In total, the system comprised 64 000 atoms. We obtained the molecular mechanics (MM) parameters for the DUE-MDA ligand from the gaff2 force field,^38^ and the MM parameters for the UMG-SP2 enzyme from the ff14SB force field.^39^ On the other hand, the atomic charges of the ligand were derived from a restrained electrostatic potential fitting^40^ performed at the HF/6-31G(d) level of theory with the Gaussian 09 software.

We employed the GROMACS software (version 2021.5)^41,42^ to minimize the energy of the assembled system *via* a three-step protocol that used the steepest descent algorithm.^43^ During the first minimization step, all water molecules were minimized. In the second step, the backbone atoms of UMG-SP2 were restrained while the remaining atoms of the system were minimized; in the final step, the whole system was minimized. The final minimized UMG-SP2 structure exhibited an RMSD of 0.38 Å (all enzyme non-hydrogen atoms were considered in the calculation) compared to the original crystallographic structure.

We then performed a classical molecular dynamics (MD) simulation to assess the overall stability of the UMG-SP2:DUE-MDA complex and to gather a structure that should correspond to a catalytically competent conformation. Throughout the entire MD protocol, all bonds involving hydrogen atoms were maintained fixed using the LINCS algorithm,^44^ which permitted the use of an integration time of 2 fs. The non-bonded interactions were explicitly calculated if under the cutoff of 10 Å, beyond which the Particle-Mesh Ewald scheme^45^ was applied to treat non-bonded Coulomb interactions. The system was initially heated to 29 °C for 100 ps at constant volume conditions (*NVT* ensemble), which was accomplished by using the V-rescale thermostat^46^ and by randomly generating initial velocities according to a Maxwell distribution. During this phase, all solute atoms were kept restrained. This heating phase was followed by a 2 ns-long *NPT* phase, in which the density of the solvent was relaxed at 1 bar and 29 °C, using the V-rescale thermostat and the Berendsen barostat.^47^ Again, all solute atoms were kept fixed while the solvent was allowed to equilibrate.

Subsequently, we performed a 100 ns-long *NPT* phase to equilibrate the overall structure of the UMG-SP2 enzyme at 29 °C and 1 bar, while preserving the geometry of the active site residues (*i.e.*, Lys91, Ser166_*cis*_, Ile187, Gly188, and Ser190_nuc_) and the carbonyl group of the ligand's urethane bond with positional restraints. We followed this initial run with a second 100 ns-long *NPT* equilibration, which was conducted with fewer positional restraints (those affecting Ile187 and Gly188 were released). Finally, we carried out a 100 ns-long *NPT* production run without any restraints, controlling the temperature and pressure with the V-rescale thermostat and the Parrinello–Rahman barostat.^48^ During this stage, we saved the configurations of the system every 200 ps, and followed five distances throughout the production phase (Fig. S1†). We used them as selection criteria to choose a catalytically competent UMG-SP2:DUE-MDA conformation: H_γ_(Ser190_nuc_)–O_γ_(Ser166_*cis*_), H_γ_(Ser166_*cis*_)–N_ζ_(Lys91), O_γ_(Ser190_nuc_)–C_carbonyl_(DUE-MDA), O_carbonyl_(DUE-MDA)–NH_backbone_(Ile187), and O_carbonyl_(DUE-MDA)–NH_backbone_(Gly188). The first three distances are related to the putative activation/deprotonation of the catalytic Ser190_nuc_ and the nucleophilic attack it should perform on the carbonyl carbon of the DUE-MDA's target urethane bond, respectively. The last two distances, calculated between two backbone amides and the ligand's carbonyl group, can be related to a successful accommodation of the target urethane bond in the cavity that hypothetically can act as an oxyanion hole throughout the enzymatic reaction. Configurations that exhibited interatomic distances inferior to 3.5 Å for O_γ_(Ser190_nuc_)–C_carbonyl_(DUE-MDA), and under 2.5 Å for the remaining four metrics were considered as being catalytically competent and potential candidates for the final UMG-SP2:DUE-MDA model. Approximately 32% of all configurations fulfilled such criteria. These configurations were ranked in ascending order of the sum of the five distances, and the top-ranked configuration (*i.e.*, the one with the smallest sum) was selected for QM/MM calculations, excluding those obtained during the first 20 ns of the production phase to ensure the use of a properly equilibrated structure.

### QM/MM calculations

**Fig. 1:**
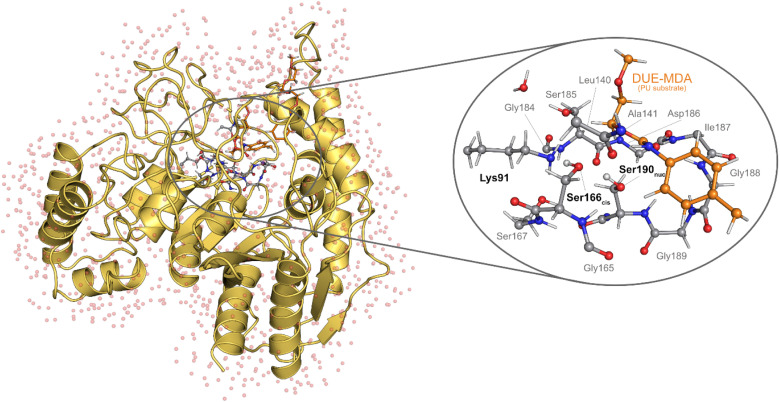
The QM/MM model used to study the catalytic mechanism of UMG-SP2. (Left) Cartoon representation of the UMG-SP2:DUE-MDA reactant structure (9929 atoms). The active site residues are represented as grey sticks, whereas the DUE-MDA substrate is shown as orange sticks. Water molecules are represented in red transparent spheres (hydrogen atoms are not shown for clarity purposes). (Right) Close-up of the QM region, composed of 126 atoms, shown in ball-and-stick representation. The PU substrate (DUE-MDA) is colored in orange. The Ser190_nuc_-Ser166_*cis*_-Lys91 catalytic triad is labeled in bold.

We built the QM/MM model based on the structure gathered from the MD simulation, after removing the Na^+^ counterions and most of the solvent water molecules. The final QM/MM model encompassed the following selection: the entire UMG-SP2 enzyme, the complete DUE-MDA ligand, all water molecules within a 3 Å radius of the enzyme, and all water molecules within 6 Å of both the active site (Lys91, Ser166_*cis*_, Ile187, Gly188, and Ser190_nuc_) and the DUE-MDA ligand. We used this QM/MM model, with 9929 atoms, to study the catalytic mechanism of the acylation reaction (STAGE 1). The truncated system was split into two regions: quantum mechanics (QM) and molecular mechanics (MM). We treated the QM region with density-functional theory (DFT) and included the most relevant atoms for the reaction to be studied (Fig. 1), *i.e.*: the complete Ser166_*cis*_, Ser185, Gly188, Gly189, and Ser190_nuc_ residues; the complete side chain of Lys91; the complete Ser167, except its backbone carbonyl group; the backbone of Asp186 and Ile187; the backbone carbonyl groups of Gly165 and Gly184; the backbone amide groups of Ala141 and Ile191; the backbone of Leu140, except its amide group; a selection of 28 atoms of the DUE-MDA ligand, which includes the target urethane bond; and a single water molecule located nearby Ser185. In total, this region comprised 126 atoms, while the remaining 9803 atoms were included in the MM region and described at the ff14SB level of theory.

We continued our study using the ONIOM subtractive scheme^49^ with electrostatic embedding in all QM/MM calculations, as implemented in the Gaussian 09 software. The valences of the bonds that crossed the boundary between the QM and MM layers were saturated with hydrogen atoms using the link-atom approach.^50,51^ All of the solvent molecules located in the MM region were frozen using the molUP plugin for the VMD software.^52,53^ The QM/MM model was initially optimized and later submitted to linear transit scans along putative reaction coordinates to investigate the steps underlying the catalytic mechanism. The linear transit scans' maxima were considered guesses for subsequent full transition state (TS) optimizations. Then, resorting to Intrinsic Reaction Coordinate (IRC) calculations, we obtained the minima associated with the optimized TSs, and their structure was subsequently optimized. We verified the nature of all stationary states as either TS (single imaginary frequency) or minima (absence of imaginary frequencies) with vibrational frequency calculations. All geometry optimizations, IRC, and vibrational frequency calculations were carried out using the B3LYP density functional^54,55^ and the 6-31G(d) basis set. Single-point QM/MM energy calculations were conducted at the B3LYP/6-311+G(2d,2p)-D3(BJ):ff14SB level of theory in the fully optimized stationary states. Grimme's D3 dispersion with Becke–Johnson damping^56^ was included in all calculations, as implemented in the Gaussian 09 software. The zero-point energy, as well as the thermal and entropic contributions to the Gibbs energy (calculated with the particle in a box/rigid rotor/harmonic oscillator formalism) were subsequently added to the final electronic energies of each stationary state to yield the corresponding Gibbs energy. Only vibrational temperatures larger than 120 K (≈100 cm^−1^) were considered for the calculation of entropic and enthalpic corrections, as validated elsewhere.^57^

To determine whether the calculated Gibbs free energy profile is independent of the selected density functional, we conducted single-point calculations on the isolated QM layer of each stationary point using ORCA 4.2.1 software.^58^ We employed B3LYP/6-311+G(2d,2p)-D3(BJ) and three additional theoretical methods: PWPB95, DSD-PBEB95, and SCS-MP2, all with the def2-TZVPP basis set. The energy difference between the three theoretical methods and B3LYP was then added as a correction to the QM/MM free energies previously calculated with Gaussian 09, yielding the final Gibbs free energies, which are presented and discussed in Table S2 and Fig. S4.†

We studied the catalytic mechanism of the deacylation reaction (STAGE 2) using a similar procedure. We built the reactant state of the deacylation stage from the product of the acylation reaction after removing the leaving group, *i.e.*, the triethylene glycol monomethyl ether. The QM/MM system used to study the deacylation reaction comprised 9905 atoms, among which 117 formed the QM layer, and the remaining 9788 atoms were included in the MM region.

Finally, we obtained the reactant structure to start exploring the reactional state, from and after optimizing the assembled structure.

### Per-residue contribution for the activation energy

To improve the catalytic efficiency of UMG-SP2 towards PU substrates, we performed an energy reassessment study to evaluate the contribution of the surrounding MM residues to the reaction energetic barrier. We used the optimized structures of the associated stationary points for the rate-limiting step. We subjected the given stationary points to single-point energy calculations, each with a targeted MM residue deleted. This protocol was applied to a total of 149 residues, with all calculations being carried out at the B3LYP-D3BJ/6-311+G(2d,2p):ff14SB level of theory. The energy contribution of each residue to the barrier was given by the difference between the barrier obtained with the given residue deleted and the wild-type barrier. Therefore, a positive difference indicated that the residue increased the energetic barrier, whilst a negative difference indicated that the residue decreased the energetic barrier. This allows for mapping the energy that each MM residue contributes to the barrier. Moreover, the residues which increase the energy barrier are the most promising mutational targets. It is important to note that our approach relies on single-point energy calculations, which do not account for potential structural rearrangements. Consequently, we strategically propose mutations under the assumption that they will not induce significant structural changes that could alter the enzyme's catalytic efficiency.

## Results and discussion

We retrieved the enzyme:ligand complex used in the mechanistic studies from the 100 ns-long MD simulation performed in this work. Throughout this simulation, the overall fold of UMG-SP2 remained stable, as shown by the RMSD analysis of the protein (Fig. S2†) and by the average RMSD for the enzyme's backbone of 1.29 ± 0.09 Å, considering the minimized structure as reference. Within this context, the docked DUE-MDA ligand also remained well-positioned for a catalytic reaction to occur: on average, the carbonyl oxygen of the target urethane bond remained at, respectively, 2.33 ± 0.48 Å and 2.13 ± 0.37 Å of the proton of the backbone amides of Ile187 and Gly188, presumed to form the oxyanion hole; the carbonyl carbon of the same urethane bond remained, on average, at 3.22 ± 0.17 Å from the O_γ_ atom of Ser190_nuc_, the nucleophilic species.

Further analysis revealed that the RMSD of the DUE-MDA ligand exhibited larger fluctuations compared to that of the enzyme. This disparity reflects the intrinsic flexibility of DUE-MDA, whose polyether tails possess high conformational freedom due to multiple rotatable bonds. Despite these fluctuations, the target urethane bond region of the substrate remained firmly lodged in the active site, with minimal deviation from the catalytically relevant positioning. Meanwhile, the enzyme displayed only minor loop movements, which did not alter the active site architecture or the binding of the substrate.

A UMG-SP2:DUE-MDA conformation was selected from the MD simulation according to a set of distance-based criteria (methods section). The structural alignment of the chosen conformation and the original X-ray structure of UMG-SP2^26^ (Fig. S3†) revealed that the two structures are very similar, both in their overall fold (backbone RMSD of 1.25 Å) and in the organization of the active site region (all-atom RMSD of 0.60 Å for the Lys91, Ser166_*cis*_, Ser167, Ser185, Ile187, Gly188, and Ser190_nuc_ set of residues). Together, these findings highlight the quality and stability of the assembled UMG-SP2:DUE-MDA model.

### STAGE 1: enzyme acylation and cleavage of the urethane bond (ester part)

**Fig. 2:**
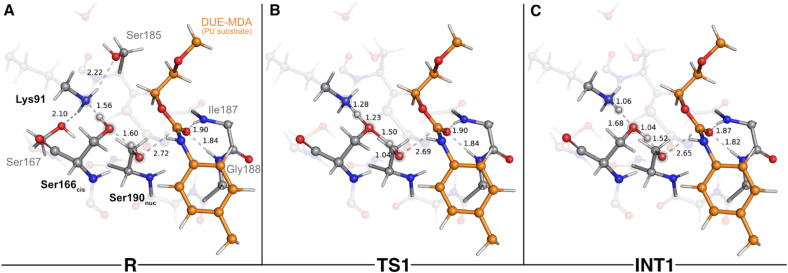
Optimized structures of the first catalytic step stationary states of UMG-SP2 (activation of Ser190_nuc_). “R”, “TS1”, and “INT1” stand for reactant (A), first transition state (B), and first intermediate (C), respectively. The most important atoms for this catalytic step are highlighted by a grey shade. The PU substrate (DUE-MDA) is colored in orange. Some QM atoms are depicted as transparent sticks for clarity purposes. Relevant distances are given in Å.

After an initial QM/MM geometry optimization, we obtained the final QM/MM model used to explore the chemical steps of the urethane bond cleavage of DUE-MDA by UMG-SP2, *i.e.* the reactant state – R. In the optimized model (Fig. 2A), the carbonyl group of the target urethane bond simultaneously rested at 1.84 and 1.90 Å of the oxyanion hole amides and at 2.72 Å of the O_γ_(Ser190_nuc_) atom; the catalytic triad residues established a hydrogen bonding network between themselves, in which Lys91 acted as the hydrogen-bond acceptor of Ser166_*cis*_ (1.56 Å), and the latter as the acceptor of a hydrogen bond of Ser190_nuc_ (1.60 Å); two serine residues, Ser167 and Ser185, were hydrogen-bonded to the catalytic Lys91 (2.10 and 2.22 Å); Ser167 and Ser185 also acted as hydrogen-bond donors to the O_backbone_ of Ser166_*cis*_ (1.89 and 2.23 Å) and a water molecule (1.88 Å), respectively (distances not shown in Fig. 2A for clarity purposes); and the O_backbone_ of Leu140 was at hydrogen-bonding distance to the NH group of the DUE-MDA's target urethane bond (distance not shown in Fig. 2A for clarity purposes). The latter was observed throughout the entire catalytic reaction (average distance of 1.98 Å).

As UMG-SP2 belongs to the amidase signature superfamily, we would expect that its catalytic reaction involved a nucleophilic attack mediated by the conserved Ser190_nuc_-Ser166_*cis*_-Lys91 triad. Our calculations revealed that before the attack on the target urethane bond of DUE-MDA takes place, the nucleophilic Ser190_nuc_ side chain hydroxyl must become activated, *i.e.* ionized. We observed that this occurs through a concerted and asynchronous reaction, in which Lys91 deprotonates Ser166_*cis*_, thus activating it so that Ser166_*cis*_ in turn can abstract a proton from the nucleophilic Ser190_nuc_. In the TS associated with this step (TS1, imaginary frequency of 909.1*i* cm^−1^), the H_γ_(Ser166_*cis*_) was nearly equidistant to O_γ_(Ser166_*cis*_) and N_ζ_(Lys91) (1.23 Å *vs.* 1.28 Å), whereas H_γ_(Ser190_nuc_) remained closer to its original position (1.04 Å to the Ser190_nuc_'s O_γ_) – Fig. 2B. Throughout the concerted proton transfer, the O_γ_(Ser190_nuc_) builds up electron density and increases its negative character, as shown by the variation in its atomic charge when moving from the reactant (−0.22 a.u.) to the first intermediate state, INT1 (−0.33 a.u.). In the latter, the Bürgi–Dunitz angle, measured from Ser190_nuc_'s side chain to the target urethane bond, adopted a value of 108°. Together, these findings reveal that the proton-transfer events rendered Ser190_nuc_ fully competent to conduct the attack on the substrate's carbonyl carbon. The calculations have shown that INT1 (Fig. 2C) corresponds to a stationary state in the potential energy surface, but not to a minimum in the Gibbs free energy profile (activation Gibbs free energy of 0.3 kcal mol^−1^ and reaction Gibbs free energy of 1.5 kcal mol^−1^), meaning that it is not a stable intermediate of the reaction cycle. This suggests that the activation of Ser190_nuc_ should be concerted with the nucleophilic attack, although occurring in an early phase of the mechanistic step. Nevertheless, as the energy difference is very small, the enzyme should be able to easily switch between the two states.

**Fig. 3:**
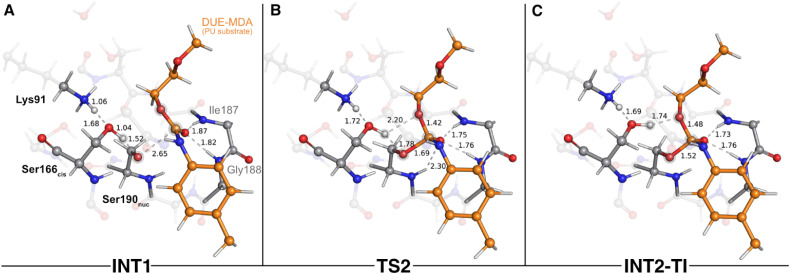
Optimized structures of the second catalytic step stationary states of UMG-SP2 (nucleophilic attack performed by Ser190_nuc_). “INT1”, “TS2”, and “INT2-TI” stand for first intermediate (A), second transition state (B), and second intermediate-tetrahedral intermediate (C), respectively. The most important atoms for this catalytic step are highlighted by a grey shade. The PU substrate (DUE-MDA) is colored in orange. Some QM atoms are depicted as transparent sticks for clarity purposes. Relevant distances are given in Å.

Once activated, Ser190_nuc_ carried out the attack on the carbonyl carbon of the DUE-MDA's urethane bond (Fig. 3). This reaction exhibited an activation Gibbs free energy (Δ*G*^‡^) of 17.4 kcal mol^−1^, and it was endergonic in 16.0 kcal mol^−1^. The TS of this catalytic step was characterized by an imaginary frequency of 151.1*i* cm^−1^ (TS2 – Fig. 3B) that was mostly associated with the stretching of the forming O_γ_(Ser190_nuc_)–C_carbonyl_(DUE-MDA) bond.

As O_γ_(Ser190_nuc_) approached the substrate's carbonyl carbon (1.69 Å in TS2), the negative character of the atoms that comprise the urethane bond increased, specifically that of O_carbonyl_ atom (DUE-MDA), which changed from −0.28 a.u. (reactant) to −0.32 a.u. (TS2). The negative charge that built up on the carbonyl group was stabilized by the oxyanion hole backbone amide groups of Ile187 and Gly188 (*ca.* 1.76 Å in TS2) and, to a lower extent, by the backbone amide group of the Ser190_nuc_ nucleophile (2.30 Å in TS2). The calculations revealed that the product of this step corresponded to a tetrahedral intermediate (INT2-TI – Fig. 3C), in which the attacking Ser190_nuc_ was covalently bound to the DUE-MDA substrate (1.52 Å *vs.* 2.72 Å in the reactant state). In this stationary state, the substrate's carbonyl C–O bond exhibited a bond length (1.30 Å) that fell between those typical of single and double bonds, evidencing the increased negative character of this group. Consequently, it was in INT2-TI where the interactions with the oxyanion hole amide groups were stronger.

Throughout the nucleophilic attack, the ion–dipole interaction between Ser190_nuc_ and Ser166_*cis*_ became progressively weaker until it was lost in INT2-TI (Fig. 3C). This occurred concomitantly to the establishment of a new hydrogen bond between Ser166_*cis*_ and the O_ester_ of the urethane bond under attack (1.74 Å in INT2-TI). As a result, Ser166_*cis*_ moved much closer to the urethane bond's O_ester_ than to its N_amide_ (1.74 Å *vs.* 3.10 Å), thus adopting a position that should favor the protonation of O_ester_(DUE-MDA) rather than the N_amide_(DUE-MDA) when the urethane bond breaking occurred. Such a role in facilitating the leaving group's protonation by the bridging Ser_*cis*_ of Ser_nuc_-Ser_*cis*_-Lys triads was also reported for other enzymes of the amidase signature superfamily, such as the fatty acid amide hydrolase.^59^ The novelty here lies in Ser166_*cis*_ that appears to play an important role in selecting which moiety should be first released as the leaving group. Specifically, our calculations showed that Ser166_*cis*_ cleaves the ester moiety rather than the amide side of the urethane bond, as we will discuss subsequently.

**Fig. 4:**
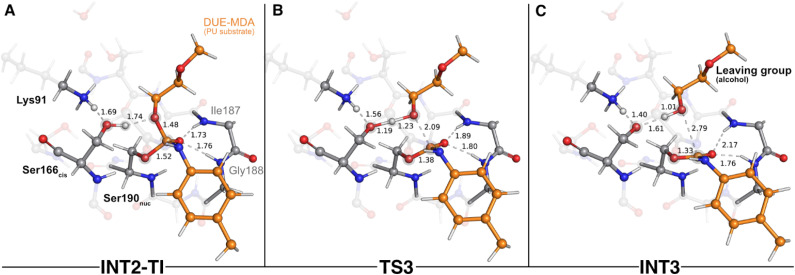
Optimized structures of the third catalytic step stationary states of UMG-SP2 (tetrahedral intermediate breakdown and urethane bond cleavage). “INT2-TI”, “TS3”, and “INT3” stand for second intermediate-tetrahedral intermediate (A), third transition state (B), and third intermediate (C), respectively. The most important atoms for this catalytic step are highlighted by a grey shade. The PU substrate atoms are colored in orange. Some QM atoms are depicted as transparent sticks for clarity purposes. Relevant distances are given in Å.

The next catalytic step involved the tetrahedral intermediate breakdown and the consequent cleavage of the urethane bond, concerted with a proton transfer from Ser166_*cis*_ to the O_ester_(DUE-MDA), yielding an alcohol-leaving group (Fig. 4). This step was characterized by a Δ*G*^‡^ of 4.8 kcal mol^−1^ and was exergonic in −3.2 kcal mol^−1^.

As the reaction proceeded from INT2-TI to TS3 (Fig. 4B), the O_ester_(DUE-MDA)–C_carbonyl_(DUE-MDA) bond elongated from 1.48 Å to 2.09 Å and the substrate's carbonyl group became more positively charged. As a result, this group re-acquired the double bond character, its interactions with the oxyanion hole became weaker, and the O_γ_(Ser190_nuc_)–C_carbonyl_(DUE-MDA) bond shortened (1.52 Å in the INT2-TI *vs.* 1.38 Å in TS3). On the other hand, O_ester_(DUE-MDA) built up electronic density (charge changed from −0.15 a.u. in INT2-TI to −0.25 a.u. in TS3) and became more prone to receive a proton from O_γ_(Ser166_*cis*_); in the TS3, the proton was virtually equidistant to both atoms (Fig. 4B). The TS of this step was characterized by an imaginary frequency at 849.6*i* cm^−1^ that was mostly dominated by the stretching of the atoms involved in the proton transfer. This indicates that, although concerted, the collapse of the tetrahedral intermediate (*i.e.*, urethane bond cleavage) preceded the proton transfer reaction, in an asynchronous event. When going from TS3 to the INT3 state (Fig. 4C), the O_ester_(DUE-MDA)–C_carbonyl_(DUE-MDA) bond became completely cleaved (2.79 Å), the proton was successfully transferred from Ser166_*cis*_ to the (now) alcohol-leaving group, and the O_γ_(Ser190_nuc_) became fully attached to the substrate's carbonyl carbon (1.33 Å). Moreover, the negative character of O_γ_(Ser166_*cis*_) increased substantially (−0.21 a.u. to −0.30 a.u.), which allowed it to become closer to the proton it previously transferred to Lys91 in the first catalytic step (1.40 Å in INT3).

**Fig. 5:**
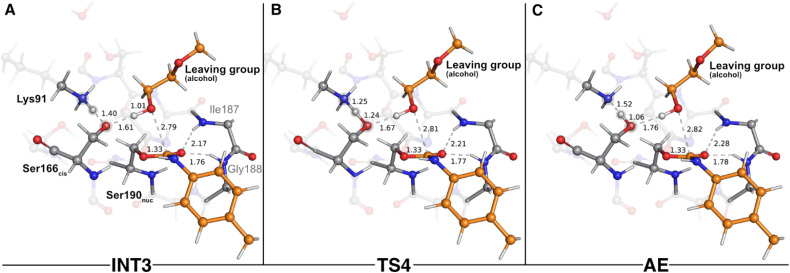
Optimized structures of the fourth catalytic step stationary states of UMG-SP2 (proton transfer from Lys91 to Ser166_*cis*_). “INT3”, “TS4”, and “AE” stand for third intermediate (A), fourth transition state (B), and acyl-enzyme (C), respectively. The most important atoms for this catalytic step are highlighted by a grey shade. The PU substrate atoms are colored in orange. Some QM atoms are depicted as transparent sticks for clarity purposes. Relevant distances are given in Å.

The final step of the enzyme acylation stage consisted of a return of the proton from Lys91 to Ser166_*cis*_, restoring their initial protonation state (Fig. 5). Although we managed to characterize a transition state for this reaction (TS4, 841.7*i* cm^−1^ – Fig. 5B), our QM/MM calculations showed that the activation barrier required to go from INT3 to the final acyl-enzyme state (AE – Fig. 5C) vanished upon the introduction of the thermal and entropic contributions to the Gibbs energy. This means that this catalytic step is barrierless and that once INT3 was formed, the proton transfer from Lys91 to Ser166_*cis*_ occurred spontaneously, yielding the AE with a reaction Gibbs free energy of −1.9 kcal mol^−1^. In the TS4 structure, the proton being shuttled to Ser166_*cis*_ was nearly halfway from the donor Lys91 residue (closer to Ser166_*cis*_ by 0.01 Å), and at the AE state the proton transfer was completed and both residues became neutral. The structural arrangement of these two states was virtually identical (all-atom RMSD of the QM layer atoms of 0.07 Å), which explains their resemblance in energy terms (Gibbs free energy difference of just 0.1 kcal mol^−1^).

After the acylation reaction, the resulting alcohol-leaving group, triethylene glycol monomethyl, was ready to abandon the active site, making it accessible to the solvent molecules required for the hydrolysis of the AE complex.

### STAGE 2: enzyme deacylation and complete degradation of the urethane bond

The second stage of the UMG-SP2 catalytic mechanism included the hydrolysis of the acyl-enzyme complex, product formation, and the regeneration of the Ser190_nuc_-Ser166_*cis*_-Lys91 triad. In the typical enzymatic hydrolysis of amide and ester bonds, the deacylation event is mediated by a water molecule that occupies the vacant space left by the leaving group, generated in the acylation stage.^29,30^ Hence, after removing the alcohol-leaving group, we modeled a water molecule (Wat_cat_) near the ester group of the acylated Ser190_nuc_ and subsequently performed a geometry optimization of the system. The gathered structure was used as the starting point to investigate the catalytic machinery behind the deacylation stage.

**Fig. 6:**
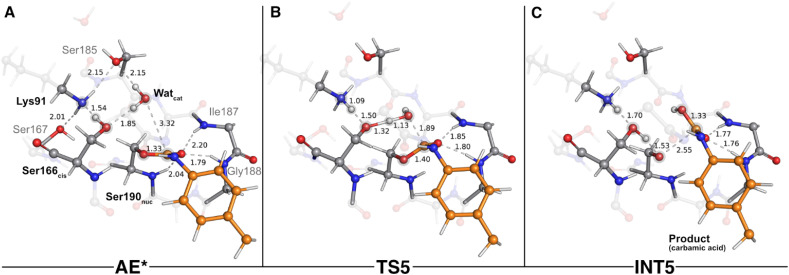
Optimized structures of the fifth catalytic step stationary states of UMG-SP2 (concerted activation of Wat_cat_ and nucleophilic attack). “AE*”, “TS5”, and “INT5” stand for acyl enzyme/reactant (stage 2, A), fifth transition state (B), and fifth intermediate (C), respectively. The most important atoms for this catalytic step are highlighted by a grey shade. The PU substrate atoms are colored in orange. Some QM atoms are depicted as transparent sticks for clarity purposes. Relevant distances are given in Å.

In the reactant state (AE* – Fig. 6A), the catalytic water rested at 3.32 Å of the substrate's carbonyl carbon, and its position was mainly dictated by the hydrogen bonds it established with Ser166_*cis*_ (1.85 Å) and with Ser185 (2.15 Å). Similarly to the reactant state of the acylation stage (R – Fig. 2A), Lys91 acted as a hydrogen bond donor to Ser167 (2.01 Å) and Ser185 (2.15 Å), while simultaneously being an acceptor of a strong hydrogen bond from Ser166_*cis*_ (1.54 Å). The substrate's carbonyl group remained lodged in the oxyanion hole cavity (at 1.79 and 2.20 Å of the amide groups) and interacted with the backbone amide of Ser190_nuc_ (2.04 Å).

The first step of the deacylation stage consisted of a concerted reaction that combined three elementary steps: a proton transfer from Ser166_*cis*_ to Lys91, a second proton transfer from Wat_cat_ to Ser166_*cis*_, and a nucleophilic attack conducted by the Wat_cat_ on the carbonyl carbon of the acylated Ser190_nuc_. This step was characterized by a Δ*G*^‡^ of 17.8 kcal mol^−1^ and was exergonic in −6.0 kcal mol^−1^. Despite the concerted nature of these catalytic events, our calculations showed that they occurred asynchronously, as discussed below. The transition state of this step (TS5 – Fig. 6B) was characterized by an imaginary frequency at 403.3*i* cm^−1^, which was largely dominated by the stretching of the O(Wat_cat_)–C_carbonyl_(DUE-MDA) and H(Wat_cat_)–O_γ_(Ser166_*cis*_) bonds. In this state, the proton transfer from Ser166_*cis*_ to Lys91 was completed (1.09 Å *vs.* 1.54 Å in the AE* state), the Wat_cat_ shared one of its hydrogen atoms with Ser166_*cis*_ (1.32 Å *vs.* 1.85 Å in the AE* state), and the nucleophilic O(Wat_cat_) rested at 1.89 Å from the target carbonyl carbon atom. This indicated that, within the same catalytic step, the activation of Ser166_*cis*_ by Lys91 preceded the remaining two events. We believe that this should be required for Ser166_*cis*_ to build up more electronic density on its side chain oxygen (charge changed from −0.24 a.u. to −0.28 a.u. from AE* to TS5) and to become more prone to activate the nucleophilic Wat_cat_.

As the reaction proceeded from TS5 to INT5 (Fig. 6C), the hydroxyl group originating from Wat_cat_ became bound to the substrate's carbonyl carbon (1.33 Å in the INT5 structure), and the bond between O_γ_(Ser190_nuc_) and the substrate was cleaved (2.55 Å in INT5 *vs.* 1.40 Å in TS5). In the acylation stage, we characterized a tetrahedral intermediate that originated from Ser190_nuc_'s nucleophilic attack on the carbonyl group of the substrate's urethane bond (INT2-TI – Fig. 3C). However, in the current stage, we did not observe the formation of a stable tetrahedral intermediate resulting from the nucleophilic attack conducted by Wat_cat_. Indeed, the IRC calculation performed to obtain INT5 from TS5 showed that the system passed through a tetrahedral geometry that immediately decayed to the INT5 state, and that, during this process, Ser190_nuc_ became deacylated and the product was released in the form of a carbamic acid (Fig. 6C). Furthermore, it also revealed that the negative character of the O_γ_(Ser190_nuc_) atom increased as Ser190_nuc_ became deacylated (charge varied from −0.13 a.u. in the TS5 to −0.32 a.u. in the INT5), which induced the establishment of a new ion–dipole interaction with Ser166_*cis*_ (1.53 Å), specifically with the hydrogen atom that Ser166_*cis*_ previously received from Wat_cat_.

**Fig. 7:**
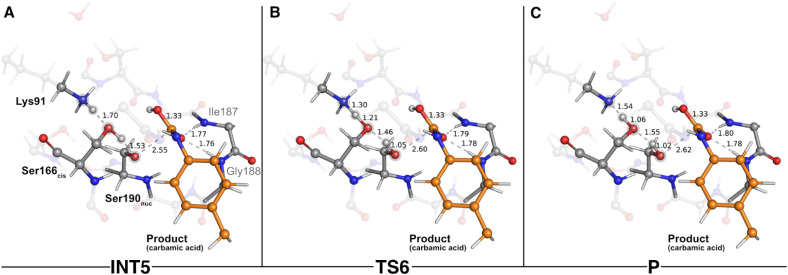
Optimized structures of sixth catalytic step stationary states of UMG-SP2 (regeneration of the catalytic triad). “INT5”, “TS6”, and “P” stand for fifth intermediate (A), sixth transition state (B), and product (C), respectively. The most important atoms for this catalytic step are highlighted by a grey shade. The PU substrate atoms are colored in orange. Some QM atoms are depicted as transparent sticks for clarity purposes. Relevant distances are given in Å.

The second step of the deacylation stage concluded the overall catalytic mechanism of UMG-SP2. It corresponded to the regeneration of the enzyme's initial state and proceeded *via* two concerted and asynchronous proton transfers between the residues of the Ser190_nuc_-Ser166_*cis*_-Lys91 triad (Fig. 7): Ser166_*cis*_ shuttled a proton to Ser190_nuc_ and accepted a proton from Lys91 (the one transferred in the opposite direction during the previous catalytic step). A closer look at the TS of this reaction (TS6 – Fig. 7B) revealed that when the proton was nearly halfway from Lys91 and Ser166_*cis*_ (1.30 Å to Lys91 and 1.21 Å to Ser166_*cis*_), the bond between O_γ_(Ser190_nuc_) and the proton transferred from Ser166_*cis*_ was already established (1.05 Å), corroborating the asynchronous nature of these events. TS6 exhibited a single imaginary frequency at 822.0*i* cm^−1^, largely dominated by the stretching of the atoms involved in the proton transfer between Lys91 and Ser166_*cis*_.

The calculations revealed that this reaction led to a state (P – Fig. 7C) that corresponded to a minimum in the potential energy surface, but not to a stable intermediate in the thermal Gibbs free energy profile (Δ*G*^‡^ = 0.5 kcal mol^−1^ and Δ*G*_reaction_ = 1.6 kcal mol^−1^). This phenomenon was also observed in the first catalytic step of the entire mechanism, where we hypothesized that the enzyme should be able to easily switch between the charged (Ser190_nuc_^−^-Ser166_*cis*_-Lys91^+^, observed in INT1) and neutral (Ser190_nuc_-Ser166_*cis*_-Lys91, observed in R) states. We believe that the same applies in this step and that both states are interchangeable.

### Overall reaction cycle of UMG-SP2

**Scheme 2:**
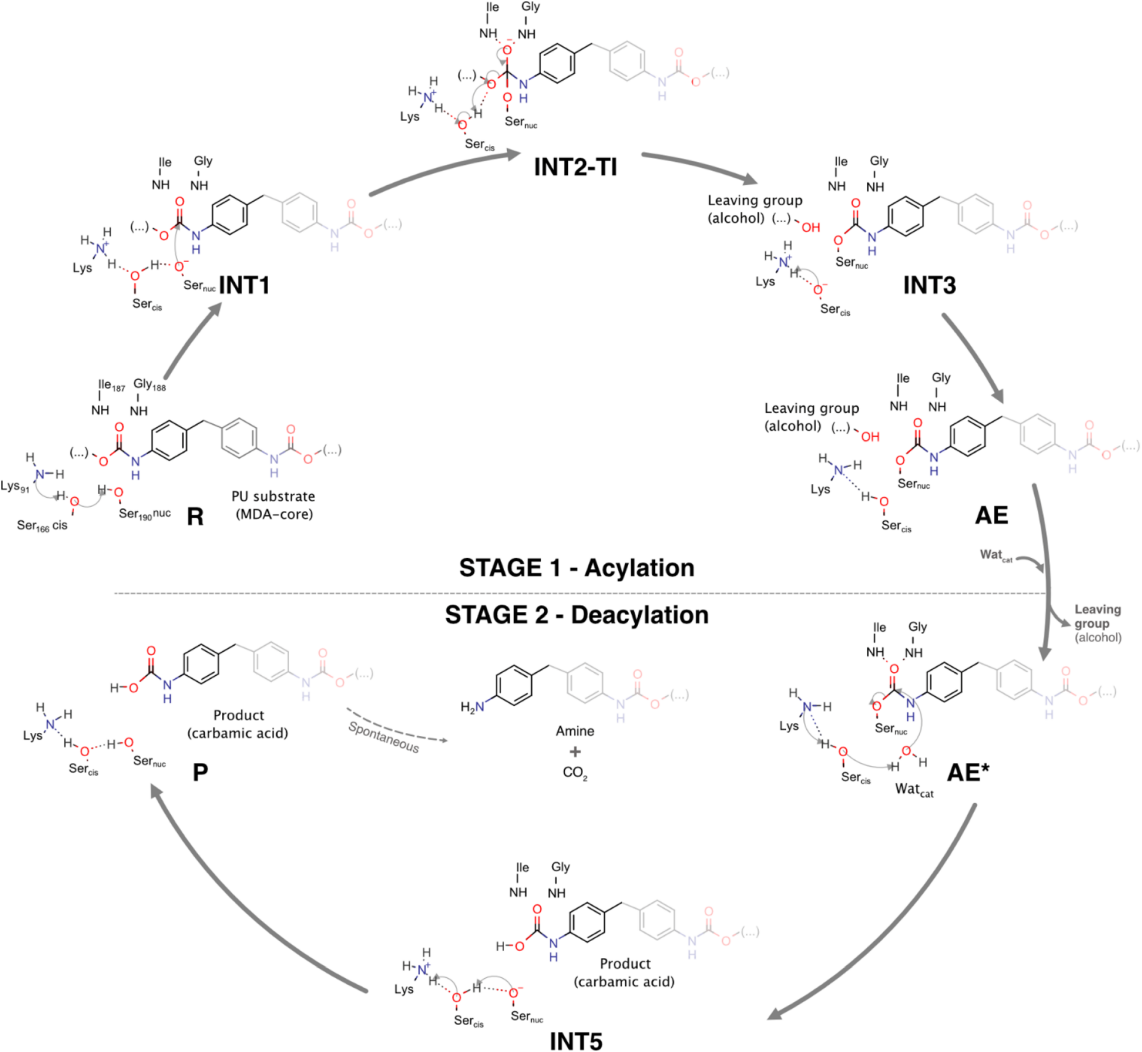
The complete catalytic mechanism for the cleavage of one urethane bond by UMG-SP2.

**Fig. 8:**
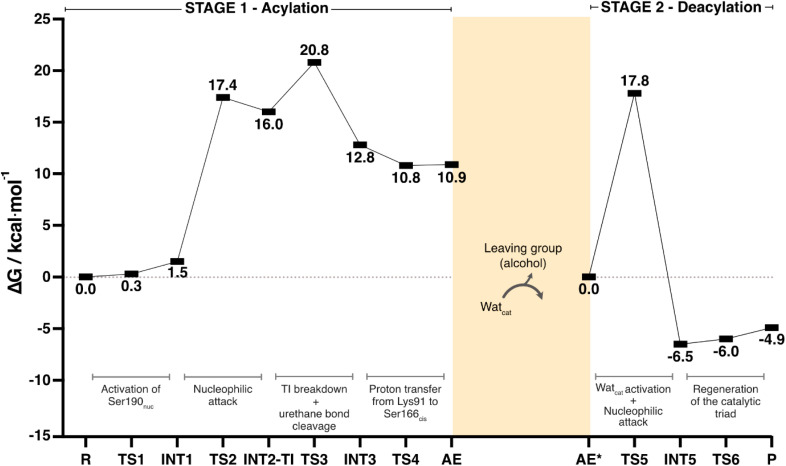
The global Gibbs free energy profile for the cleavage of one urethane bond by UMG-SP2. The presented Δ*G* values were determined at the B3LYP/6-311+G(2d,2p)-D3(BJ):ff14SB//B3LYP/6-31G(d)-D3(BJ):ff14SB level of theory and are presented in kcal mol^−1^. The energy profiles of each stage are shown separately. Connecting the Gibbs energy profiles of the two stages requires complex and often inaccurate calculations of the Gibbs energy for the alcohol-leaving group dissociation and active site solvation, that being why we adopted the current representation. Each mechanistic step is indicated in the bottom part of the plot, in dark grey.

Scheme 2 and Fig. 8 depict the complete catalytic mechanism and the global Gibbs free energy profile for the cleavage of one urethane bond of the PU substrate DUE-MDA by UMG-SP2.

The catalytic reaction occurred in two well-defined stages: in STAGE 1, the enzyme becomes covalently bound to the PU substrate, and an alcohol-leaving group is released; in STAGE 2, the enzyme:ligand covalent adduct is hydrolyzed by a catalytic water molecule and the product is released in the form of a carbamic acid (Scheme 2).

According to Fig. 8, the complete catalytic reaction involves a Gibbs activation free energy of 20.8 kcal mol^−1^ for the highest barrier identified. While this represents the primary barrier characterized in our study, additional phenomena such as product dissociation and solvent diffusion, which occur under real-world conditions, may also contribute to the overall reaction energetics. Even though the turnover rate for this enzyme has not been reported, we find reassurance in the fact that the theoretically calculated energy barrier laid within the range of values (∼13–23 kcal mol^−1^) corresponding to the experimental catalytic rates of most known hydrolases.^60^ The rate-limiting transition state of the overall reaction (TS3) corresponded to the concerted reaction that combined the tetrahedral intermediate breakdown and the cleavage of the substrate's urethane bond by its ester moiety with the subsequent formation of the alcohol-leaving group.

Our calculations revealed that a carbamic acid product resulted from the urethane bond cleavage of DUE-MDA by UMG-SP2. Carbamic acids are known to be unstable at room temperature and to quickly decompose to form an amine and carbon dioxide (CO_2_).^61,62^ Therefore, we believe the product we characterized should quickly eliminate CO_2_ and give rise to a monosubstituted MDA-urethane compound, specifically mono-urethane ethylene 4,4′-methylenedianiline (MUE-MDA). A second catalytic cycle of UMG-SP2 would complete the cleavage of the remaining urethane bond of MUE-MDA, releasing the alcohol-leaving group, triethylene glycol monomethyl, and the carbamic acid form of 4,4′-methylenedianiline (MDA). Again, the latter should quickly decompose into CO_2_ and MDA (the amine), which, interestingly, was detected as the end product of UMG-SP1's activity on a synthetic MDA diisocyanate-based thermoplastic polyester-PU.^25^

### Contribution of individual residues to the activation energy

Even though the UMG-SP2 has shown considerable activity towards dicarbamates (PU monomers), there is still room to enhance its efficiency with this substrate. The catalytic efficiency is influenced by *k*_cat_ and *K*_M_, and we focus here on *k*_cat_, which is typically calculated with greater accuracy and is a primary target in directed evolution. As it is known that *k*_cat_ is related to the energy barrier, we calculated the energy contribution of each surrounding MM residue to the energy barrier to identify mutations that could enhance the catalytic efficiency by improving the *k*_cat_, rate-limiting steps involving significant electron density rearrangements are stabilized or destabilized by surrounding charged residues. This effect is dependent on the nature of the active site macrodipole and the positioning of these residues within the active site.^63–65^

By definition, residues that destabilize the rate-limiting TS (TS3) in relation to the reactant state increase the energy barrier, while those that stabilize it will decrease the barrier. Based on the nature of TS3 and the reactant state, we predict that positive residues closer to the O_ester_(DUE-MDA) than to the O_γ_(Ser190_nuc_) will stabilize the TS3 in relation to the reactant state. Conversely, positive residues nearer the O_γ_(Ser190_nuc_) will do the opposite. For negative residues, those closer to the O_γ_(Ser190_nuc_) are expected to stabilize TS3 in relation to the reactant state, while those nearer to the O_ester_(DUE-MDA) will do the opposite. The residues that destabilize TS3 are the most promising targets for mutation.

**Fig. 9:**
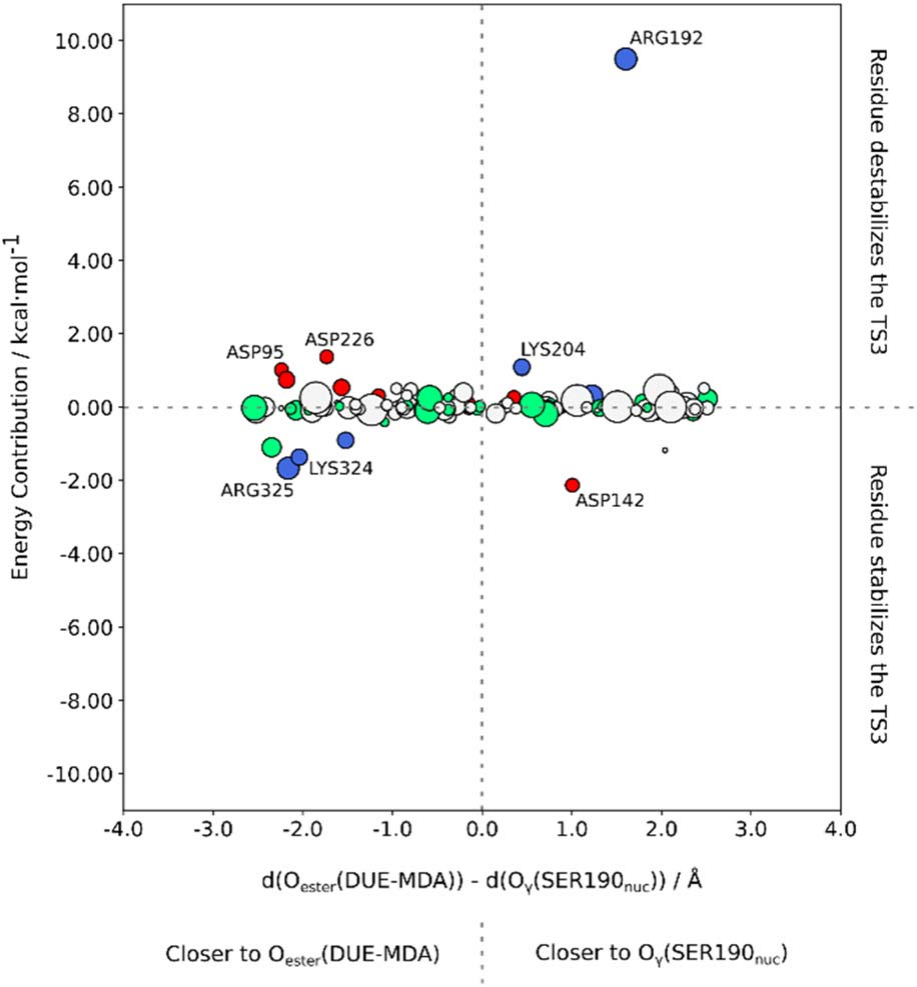
The impact of each surrounding MM residue on the reaction energy barrier as a function of the difference between the distance of the given MM residue to the positive (O_γ_(Ser190_nuc_)) and the negative sides (O_ester_(DUE-MDA)) of the TS3 macrodipole. Positive residues are colored blue, while negative residues are colored red. Polar residues are colored green, and hydrophobic residues are colored white. Only the residues that have a relevant energy contribution (*e.g.*, |1.0| kcal mol^−1^) are identified. The top half of the graph shows the residues that destabilize TS3, *i.e.* the most promising targets for mutation.

Fig. 9 shows the impact of each deleted MM residue on the energy barrier. Additionally, the energy contribution of each residue is plotted as a function of the difference between the distance to the positive side of the TS3 macrodipole (O_γ_(Ser190_nuc_)) and the distance to the negative side (O_ester_(DUE-MDA)). For this measurement, we assigned a reference carbon atom for each type of residue (Table S4†).

We observed that most residues with a significant energy contribution to the barrier were charged. Additionally, the majority of those charged residues were closer to the O_ester_(DUE-MDA) than to O_γ_(Ser190_nuc_). Furthermore, most negative residues were located closer to the negative side of the TS3 macrodipole. Consistent with our prediction, residues Asp95 and Asp226 destabilized TS3, leading to an increase of the energy barrier. Since these residues were closer to the negative side of the TS3 macrodipole, we suggest mutating them to polar residues (*e.g.*, Asn) for a more conservative strategy, or to positive residues (Lys or Arg) for a structurally riskier approach. Conversely, Asp142 was closer to O_γ_(Ser190_nuc_) than to O_ester_(DUE-MDA), placing it closer to the positive side of the TS3 macrodipole, thereby lowering the energy barrier.

We also predicted that positive residues closer to the O_ester_(DUE-MDA) than to O_γ_(Ser190_nuc_) stabilize the TS3, contributing to a lower energy barrier. In line with our rationale, Lys324 and Arg325, located near the negative side of the macrodipole, stabilized TS3. On the other hand, we predicted that positive residues closer to the O_γ_(Ser190_nuc_) than to O_ester_(DUE-MDA) destabilize TS3. Accordingly, Arg192 and Lys204, being near the positive side of the TS3 macrodipole, destabilized TS3. Notably, Arg192 significantly increased the energy barrier, making it the most promising mutational target. Arg192 is surrounded by neutral residues, with a lack of strong electrostatic interactions in its vicinity. The absence of stabilizing interactions for the positive charge resulted in the destabilization of its surroundings. This charged residue is close to the backbone of Ser190_nuc_, Ile187, and Gly188, which are key residues for the reaction, meaning their stability is essential. The presence of a nearby unstable positive charge in the vicinity of these residues led to their destabilization, consequently destabilizing TS3. Hence, Arg192 should be mutated to a polar or hydrophobic residue (*e.g.*, Gln, His, or Met) to stabilize the neutral residue network. Concerning Lys204, as it is closer to the positive side of the TS3 macrodipole, we propose mutating it to a neutral residue (*e.g.*, Gln or Met) for a more conservative strategy or to a negative residue (*e.g.*, Asp or Glu) for a more aggressive strategy.

Based on our method, we believe that mutating the four aforementioned targets should stabilize the TS3 macrodipole. This stabilizing effect will lower the energy barrier, leading to an increase in the *k*_cat_ term, achieving our goal of improving the UMG-SP2 hydrolysis of PU substrates.

## Conclusion

In this work, we investigated the hydrolysis of a model substrate of PU (DUE-MDA), catalyzed by the metagenome-derived UMG-SP2 enzyme. We employed computational methods to unveil, with atomic detail, the catalytic machinery behind this enzymatic reaction and determined its overall Gibbs free energy profile (Fig. 8).

Our calculations demonstrated that UMG-SP2 cleaves urethane bonds in two mechanistic stages, acylation and deacylation, and that the first comprises the rate-limiting step with an overall Δ*G*^‡^ of 20.8 kcal mol^−1^. The catalytic cycle culminates with the release of the final product in the form of a carbamic acid (Fig. 8A). Notably, the enzyme does not fully degrade the target urethane bond, but rather cleaves its ester moiety (esterase-like activity), leading to a highly unstable product. The latter should, in an enzyme-independent manner, rapidly decompose to form CO_2_ and an amine, causing the complete degradation of the urethane bond.

The QM/MM calculations shed light on the specific role of the active site residues during the urethane bond hydrolysis. They corroborated that Ser190_nuc_ is the nucleophile, whose reactivity is controlled by the remaining two residues of the catalytic triad (Lys91 and Ser166_*cis*_). Lys91 establishes persistent hydrogen bond interactions with two nearby residues (Ser167 and Ser185) and, by deprotonation/protonation events, respectively activates/deactivates the bridging Ser166_*cis*_. The latter acts as a catalytic base, activating the nucleophilic Ser190_nuc_, and is also responsible for the protonation of the substrate's leaving group. In addition, Ser166_*cis*_ plays a major role in selecting which moiety (ester *vs.* amine) should be released as the leaving group. The structural rearrangement of Ser166_*cis*_'s side chain during the nucleophilic attack by Ser190_nuc_ facilitates the proton transfer to O_ester_(DUE-MDA) and the subsequent urethane bond cleavage by its ester side. Throughout these events, the enzyme's oxyanion hole accommodates the urethane bond's carbonyl and stabilizes the tetrahedral reaction intermediates.

Finally, we have identified four mutational targets predicted to stabilize the TS3 macrodipole, which are expected to decrease the energy barrier and enhance the catalytic efficiency of UMG-SP2 for urethane bond cleavage.

Overall, the findings reported herein offer valuable insight into the catalytic mechanism underlying the hydrolysis of PU substrates by UMG-SP2. We hypothesize that, to some extent, many of the reported phenomena may be common to the other two metagenomic urethanases (UMG-SP1 and UMG-SP3) and to other enzymes of the amidase signature superfamily, although further studies are needed to corroborate this assumption. We hope this work encourages future research on the enzymatic depolymerization of PU that aims to address the environmental issues arising from the widespread use of plastics.

## Author contributions

P. P., P. A. F., and M. J. R. designed research; P. P. and L. T. performed research and analyzed data. All authors contributed to the writing of the manuscript. The paper has been approved in its final form by all authors.

## Conflicts of interest

The authors declare that they have no competing interests.

## Supplementary Material

SC-016-D4SC06688J-s001

SC-016-D4SC06688J-s002

## Data Availability

The data supporting this article have been included within the manuscript and as part of the ESI.† Additional data supporting the findings of this study are available from the corresponding author upon request.
