# Supplementary material for: Unveiling the enzymatic pathway of UMG-SP2 urethanase: insights into polyurethane degradation at the atomic level
Source: Chem Sci. 2024 Dec 18;16(5):2437–52. doi: 10.1039/d4sc06688j (PMC11708778; doi:10.1039/d4sc06688j)
Supplement: SC-016-D4SC06688J-s002 [file SC-016-D4SC06688J-s002.pdf]

Supplementary Information (SI) for Chemical Science.
This journal is © The Royal Society of Chemistry 2024

SUPPORTING INFORMATION

Unveiling the Enzymatic Pathway of UMG-SP2 Urethanase: Insights into Polyurethane Degradation at the Atomic Level

Paiva, P.[1,2], Teixeira, L.M.C.[1,2], Wei, R.[3], Liu, W.[4], Weber, G.[5], Morth, J.P.[2,6], Westh, P.[2,6], Petersen, A.R.[2,7] , Johansen, M.B.[2,7], Sommerfeldt, A.[2,7], Sandahl, A.[2,7], Otzen, D. E.[2,8], Fernandes, P.A.[1,2], and Ramos, M.J.[1,2] *

[1] LAQV@REQUIMTE, Departamento de Química e Bioquímica, Faculdade de Ciências, Universidade do Porto, Rua do Campo Alegre s/n, 4169-007, Porto, Portugal.

[2] EnZync Center for Enzymatic Deconstruction of Thermoset Plastics.

[3] Junior Research Group Plastic Biodegradation, Department of Biotechnology & Enzyme Catalysis, Institute of Biochemistry, University of Greifswald, Felix-Hausdorff-Str. 8, 17489 Greifswald, Germany.

[4] Tianjin Institute of Industrial Biotechnology, Chinese Academy of Sciences, 32 West Seventh Avenue, Tianjin Airport Economic Area, Tianjin 300308, China.

[5] Macromolecular Crystallography, Helmholtz-Zentrum Berlin, Alber-Einstein-Straße 15, 12489 Berlin, Germany.

[6] Department of Biotechnology and Biomedicine, Technical University of Denmark, Søltofts Plads, DK-2800, Kongens Lyngby, Denmark.

[7] Teknologisk Institut, DK-8000, Kongsvang Alle 29, Aarhus, Denmark.

[8] Interdisciplinary Nanoscience Center (iNANO). Aarhus University, Gustav Wieds Vej 14, DK - 8000 Aarhus, Denmark.

*email: mjramos@fc.up.pt

UMG-SP2:DUE-MDA complex modeling

Table S1. List of predicted pK_a values for titratable residues estimated by PROPKA 3.5.0.

Residue	pK_a	pK_a-model
**Asp40**	2.6	3.8
**Asp50**	5.3	3.8
**Asp60**	3.8	3.8
**Asp62**	4.0	3.8
**Asp66**	3.8	3.8
**Asp72**	3.3	3.8
**Asp95**	5.0	3.8
**Asp110**	3.1	3.8
**Asp116**	3.0	3.8
**Asp142**	3.6	3.8
**Asp158**	2.5	3.8
**Asp186**	1.9	3.8
**Asp208**	3.3	3.8
**Asp221**	3.8	3.8
**Asp226**	5.2	3.8
**Asp239**	5.5	3.8
**Asp245**	3.4	3.8
**Asp277**	3.9	3.8
**Asp304**	4.0	3.8
**Asp307**	3.2	3.8
**Asp314**	4.2	3.8
**Asp345**	4.0	3.8
**Asp346**	3.3	3.8
**Asp355**	4.6	3.8
**Asp362**	2.9	3.8
**Asp378**	3.8	3.8
**Asp381**	3.4	3.8
**Asp390**	3.0	3.8
**Asp421**	2.9	3.8
**Asp438**	5.0	3.8
**Asp440**	4.5	3.8
**Asp453**	4.1	3.8
**Glu18**	4.0	4.5
**Glu23**	3.8	4.5
**Glu37**	3.9	4.5
**Glu47**	4.7	4.5
**Glu74**	4.1	4.5
**Glu92**	5.2	4.5
**Glu107**	4.3	4.5
**Glu182**	7.0	4.5

**Glu213**	4.7	4.5
**Glu273**	3.9	4.5
**Glu285**	4.8	4.5
**Glu292**	4.8	4.5
**Glu330**	4.5	4.5
**Glu333**	4.7	4.5
**Glu359**	4.8	4.5
**Glu379**	4.3	4.5
**Glu450**	4.7	4.5
**His108**	5.8	6.5
**His111**	6.4	6.5
**His157**	5.7	6.5
**His159**	6.8	6.5
**His196**	4.3	6.5
**His215**	1.6	6.5
**His271**	4.6	6.5
**His376**	5.4	6.5
**His439**	6.4	6.5
**Cys39**	11.3	9
**Cys198**	12.7	9
**Cys291**	11.5	9
**Cys366**	12.4	9
**Tyr150**	13.6	10
**Tyr183**	13.0	10
**Tyr216**	10.9	10
**Tyr315**	13.6	10
**Tyr341**	10.2	10
**Tyr408**	12.4	10
**Lys69**	10.3	10.5
**Lys81**	10.9	10.5
**Lys91**	6.1	10.5
**Lys124**	10.3	10.5
**Lys133**	8.4	10.5
**Lys204**	6.6	10.5
**Lys224**	8.1	10.5
**Lys249**	11.0	10.5
**Lys384**	10.6	10.5
**Arg45**	11.1	12.5
**Arg49**	12.8	12.5
**Arg59**	12.4	12.5
**Arg63**	13.6	12.5
**Arg65**	14.2	12.5
**Arg80**	12.1	12.5
**Arg122**	12.0	12.5

**Arg152**	12.0	12.5
**Arg161**	11.8	12.5
**Arg192**	13.0	12.5
**Arg219**	12.6	12.5
**Arg235**	10.8	12.5
**Arg256**	12.4	12.5
**Arg264**	13.6	12.5
**Arg286**	12.0	12.5
**Arg317**	13.1	12.5
**Arg325**	12.3	12.5
**Arg349**	13.3	12.5
**Arg352**	12.1	12.5
**Arg356**	12.5	12.5
**Arg385**	12.9	12.5
**Arg434**	12.4	12.5
**Arg443**	12.2	12.5
**Arg454**	12.5	12.5

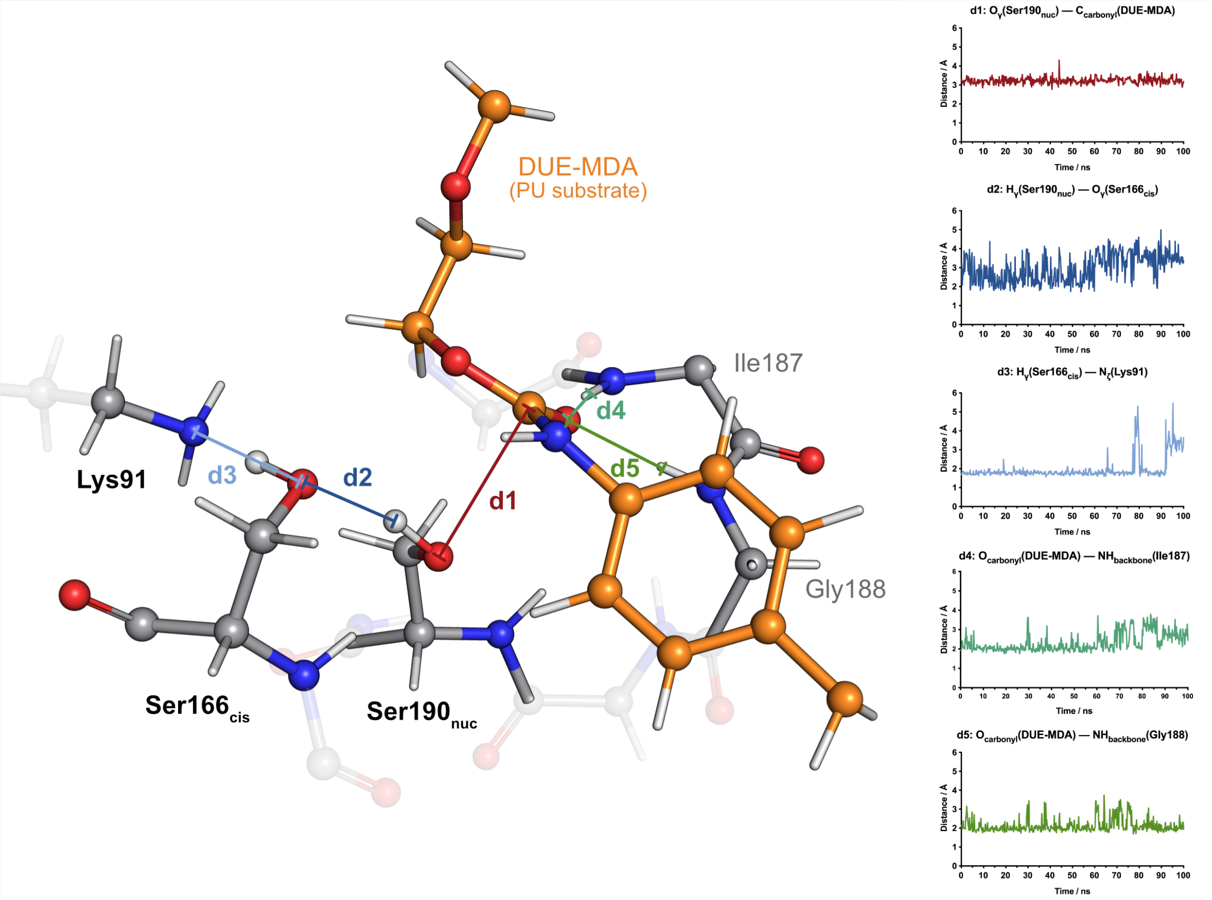

Figure S1. Catalytically relevant interatomic distances that were followed throughout the 100 ns-long production phase.

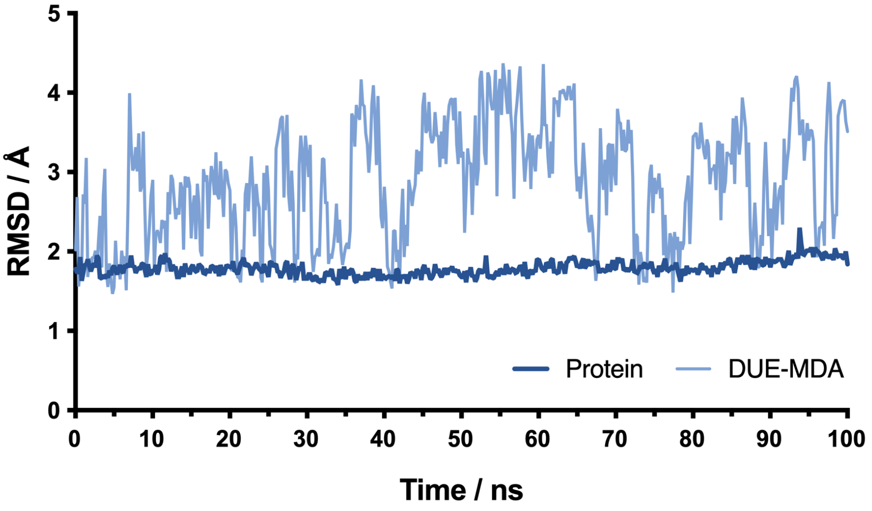

Figure S2. RMSD fluctuation of the protein (dark blue) and the DUE-MDA substrate (light blue) during the 100 ns-long production phase.

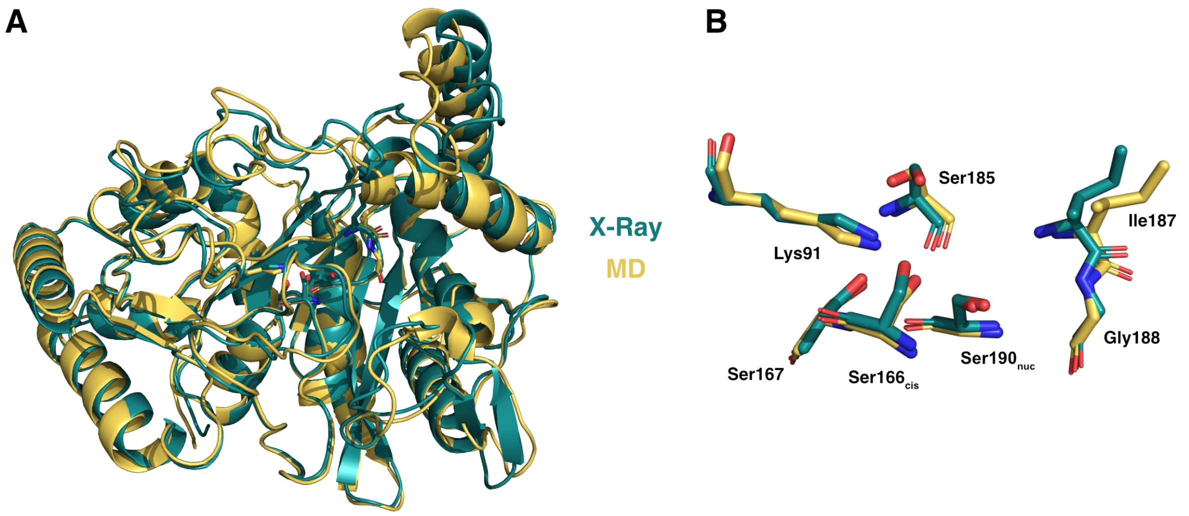

Figure S3. Structural alignment of the X-ray structure of UMG-SP2 complexed with the inhibitor phenylmethanesulfonyl fluoride (PDB ID: 8WDW) and the UMG-SP2's MD-gathered structure that was used in the QM/MM mechanistic study. Left: cartoon representation of the X-ray (teal) and MD-derived (gold) UMG-SP2 structures. The active site residues are represented as thick sticks. The phenylmethanesulfonyl fluoride inhibitor is not shown for clarity purposes. Right: close-up of the active site region, in which a selection of residues is shown as teal (x-ray) or gold (MD) sticks.

Assessing the effect of different theoretical methods on the free energy profile

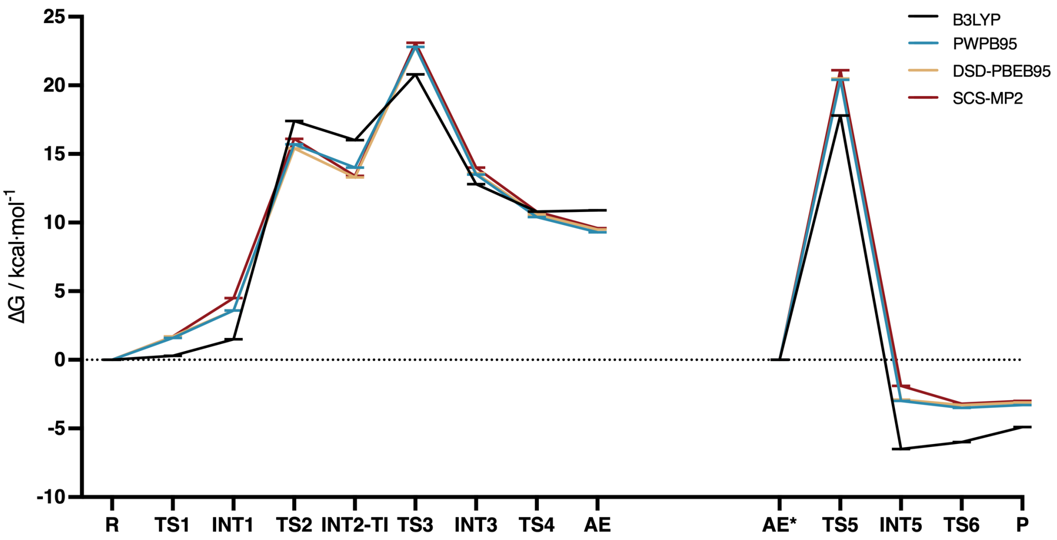

Figure S4. Free energy profiles for the UMG-SP2-catalyzed reaction, determined at different theoretical levels. The ΔG values were determined using the B3LYP/6-31G(d)-D3(BJ):*ff*14SB-optimized geometries of each stationary state. The B3LYP single-point calculations used the 6-311+G(2d,2p) basis set and the D3(BJ) dispersion correction. The single-point calculations performed with the remaining theoretical methods were conducted with the def2-TZVPP basis set. The D3(BJ) dispersion correction was contemplated in the calculations performed with the PWPB95 and DSD-PBEB95 density functionals. The values are presented in kcal·mol^{-1}.

Table S2. ΔG values, determined at different theoretical levels. The calculations were performed using the B3LYP/6-31G(d)-D3(BJ):*ff*14SB-optimized geometries of each stationary state. The B3LYP single-point calculations used the 6-311+G(2d,2p) basis set and the D3(BJ) dispersion correction. The single-point calculations performed with the remaining theoretical methods were conducted with the def2-TZVPP basis set. The D3(BJ) dispersion correction was contemplated in the calculations performed with the PWPB95 and DSD-PBEB95 density functionals. The values are presented in kcal·mol^{-1}.

Stationary State	B3LYP	PWPB95	DSD-PBEB95	SCS-MP2
**R**	0.0	0.0	0.0	0.0
**TS1**	0.3	1.6	1.7	1.7
**INT1**	1.5	3.6	3.6	4.5
**TS2**	17.4	15.7	15.4	16.1
**INT2-TI**	16.0	14.0	13.3	13.4
**TS3**	20.8	22.8	22.8	23.1
**INT3**	12.8	13.5	13.6	14.0
**TS4**	10.8	10.4	10.6	10.8
**AE**	10.9	9.3	9.5	9.6

**AE***	0.0	0.0	0.0	0.0
**TS5**	17.8	20.4	20.5	21.1
**INT5**	-6.5	-3.0	-2.9	-1.9
**TS6**	-6.0	-3.5	-3.3	-3.2
**P**	-4.9	-3.3	-3.1	-3.0

We conducted single-point calculations using three additional theoretical methods to determine whether the calculated Gibbs free energy profile is independent of the density functional used (B3LYP): the double-hybrid-meta-GGA functional PWPB95, which has 50 % Hartree-Fock exchange; the double-hybrid-meta-GGA functional DSD-PBEB95, which has 66 % Hartree-Fock exchange; and the resolution identity spin-component-scaled second-order Møller–Plesset perturbation theory (SCS-MP2).

The calculated ΔG values for the four theoretical methods were similar, suggesting that the overall Gibbs free energy landscape remained consistent. TS3 consistently remained as the rate-limiting TS of the overall reaction, with its energy fluctuating only slightly within the range of 20.8 to 23.1 kcal·mol^{-1}.

QM/MM study of the UMG-SP2 catalytic mechanism

Table S3. Most relevant interatomic distances involved in the hydrolysis of the DUE-MDA PU substrate, determined at ONIOM(B3LYP/6-31G(d)-D3(BJ):*ff*14SB level of theory. The values are presented in Å.

Stationary State	$O_γ$(Ser190$_{nuc}$) – C_{carb}(DUE-MDA)	$H_γ$ (Ser190$_{nuc}$) – $O_γ$(Ser166$_{cis}$)	$H_γ$(Ser166$_{cis}$) – $N_ζ$(Lys91)	C_{carb}(DUE-MDA) – O_{carb}(DUE-MDA)	$O_γ$(Ser167) – $N_{ζ1}$(Lys91)	$O_γ$(Ser185) – $N_{ζ1}$(Lys91)	O_{carb}(DUE-MDA) – NH(Ile187)	O_{carb}(DUE-MDA) – NH(Gly188)
**R**	2.72	1.60	1.56	1.34	2.10	2.22	1.90	1.84
**TS1**	2.69	1.50	1.28	1.34	2.00	2.16	1.90	1.84
**INT1**	2.65	1.04	1.06	1.34	1.83	1.97	1.87	1.82
**TS2**	1.69	0.99	1.05	1.42	1.82	1.88	1.75	1.76
**INT2-TI**	1.52	0.99	1.04	1.48	1.79	1.86	1.73	1.76
**TS3**	1.38	1.19	1.07	2.09	1.84	1.89	1.89	1.80
**INT3**	1.33	1.61	1.14	2.79	1.93	1.98	2.17	1.76
**TS4**	1.33	1.67	1.25	2.81	1.99	2.06	2.21	1.77
**AE**	1.33	1.76	1.52	2.82	2.10	2.18	2.28	1.78
**Stationary State**	**$O_γ$(Ser190$_{nuc}$) – C_{carb}(DUE-MDA)**	**H(Wat$_{cat}$) – $O_γ$(Ser166$_{cis}$)**	**$H_γ$(Ser166$_{cis}$) – $N_ζ$(Lys91)**	**O(Wat$_{cat}$) – C_{carb}(DUE-MDA)**	**$O_γ$(Ser167) – $N_{ζ1}$(Lys91)**	**$O_γ$(Ser185) – $N_{ζ1}$(Lys91)**	**O_{carb}(DUE-MDA) – NH(Ile187)**	**O_{carb}(DUE-MDA) – NH(Gly188)**
**AE***	1.33	1.85	1.54	3.32	2.01	2.15	2.20	1.79
**TS5**	1.40	1.32	1.09	1.89	1.88	1.93	1.85	1.80
**INT5**	2.55	1.03	1.05	1.33	1.87	1.94	1.77	1.76
**TS6**	2.60	1.46	1.30	1.33	2.05	2.13	1.79	1.78
**P**	2.62	1.55	1.54	1.33	2.13	2.19	1.80	1.78

Contribution of individual residues to the activation energy

Table S4. Reference carbon atoms. The table presents the residue-specific carbon atoms, defined as the reference atoms used to measure the distance of the given residue to the O_{ester}(DUE-MDA) and O_{γ}(Ser190$_{nuc}$).

Residue	Reference atom
**Nonpolar**	C_{α}
**Arginine**	C_{ζ}
**Asparagine**	C_{γ}
**Aspartate**	C_{γ}
**Cysteine**	C_{β}
**Glutamine**	C_{δ}
**Glutamate**	C_{δ}
**Histidine**	$C_{\delta2}$/$C_{\varepsilon1}$
**Lysine**	C_{ε}
**Methionine**	C_{β}
**Threonine**	C_{β}
**Tyrosine**	C_{β}
